# 

**DOI:** 10.3325/cmj.2011.52.746

**Published:** 2011-12

**Authors:** Srećko Gajović

**In Reply:** The *Croatian Medical Journal* strictly follows the rule that manuscripts based on unregistered clinical trials are immediately rejected (1-3).

## References

[1] Marusic A, Huic M (2008). Registration of clinical trials still moving ahead – September 2008 update to Uniform Requirements for Manuscripts Submitted to Biomedical Journals.. Croat Med J.

[2] Laine C, Horton R, DeAngelis CD, Drazen JM, Frizelle FA, Godlee F (2007). Clinical trial registration: looking back and moving ahead.. Croat Med J.

[3] De Angelis C, Drazen JM, Frizelle FA, Haug C, Hoey J, Horton R (2004). Clinical trial registration: a statement from the International Committee of Medical Journal Editors.. Croat Med J.

